# Surgical treatment of multiple breast cancer brain metastases: clinical characteristics and factors impacting postoperative survival

**DOI:** 10.1007/s11060-025-05048-3

**Published:** 2025-04-29

**Authors:** Anna Michel, Laurèl Rauschenbach, Hanah Karadachi, Meltem Gümüs, Yahya Ahmadipour, Marvin Darkwah Oppong, Christoph Pöttgen, Jörg Hense, Neriman Özkan, Karsten H. Wrede, Philipp Dammann, Ulrich Sure, Ramazan Jabbarli

**Affiliations:** 1 Department of Neurosurgery and Spine Surgery, University Hospital Essen, Hufelandstraße 55, 45147 Essen, Germany; 2 Center for Translational Neuro‑ & Behavioral Sciences (C‑TNBS), University Duisburg Essen, Essen, Germany; 3 German Cancer Consortium (DKTK) Partner Site, University Hospital Essen, 45147 Essen, Germany; 4 German Cancer Research Center (DKFZ) Division Translational Neurooncology at the West German Cancer Center (WTZ), DKTK Partner Site, University Hospital Essen, Essen, Germany; 5 Department of Radiotherapy, University Hospital Essen, Essen, Germany; 6 Department of Medical Oncology, University Hospital Essen, Essen, Germany

**Keywords:** Breast cancer, Surgical treatment, Multiple brain metastases, Adjuvant radiotherapy, Trastuzumab

## Abstract

**Purpose:**

Breast cancer (BC) is one of the most common primary tumor entities that develop brain metastases (BM) during disease progression. Multiple BM are associated with poorer prognosis, but various surgical, radiotherapeutic and systemic treatment approaches improve survival. We aimed to identify prognostic factors and evaluate the overall survival following BM surgery in patients with multiple BCBM.

**Methods:**

All metachronous metastasized female patients with resected BCBM at our institution between 2008 and 2019 were included. Data on clinical, radiologic, and histopathologic parameters were recorded and analyzed using univariate and multivariate regression models.

**Results:**

Among the 93 patients included in the final analysis, 30 individuals presented with multiple BM. Compared to patients with single BM, those with multiple BM were more likely to have infratentorial BM (adjusted odds ratio [aOR] 3.35, 95% confidence interval [CI] 1.03–10.83, *p* = 0.044), HER2(human epidermal growth factor receptor 2)-positive BC (aOR 3.93, 95% CI 1.23–12.53, *p* = 0.021) and hepatic metastases (aOR 5.86, 95% CI 1.34–25.61, *p* = 0.019). There was no significant difference in postoperative survival between individuals with multiple (median: 12.5 months) and single BM (17.0 months, *p* = 0.186). In the multivariate Cox regression analysis, adjuvant radiotherapy (adjusted hazard ratio [aHR] 5.93, 95% CI 1.06–33.26, *p* = 0.043) and trastuzumab treatment (aHR 4.95, 95% CI 1.72–14.25, *p* = 0.003) were associated with longer postoperative survival multiple BCBM patients.

**Conclusion:**

BC patients with multiple BM show remarkable postoperative survival, particularly if combined with adjuvant radiotherapy. Our data justify the surgery of multiple BCBM in patients with appropriate clinical condition and feasible location of BM.

**Supplementary Information:**

The online version contains supplementary material available at 10.1007/s11060-025-05048-3.

## Introduction

Breast cancer (BC) is one of the most common cancer entities in women, following lung cancer [1]. Despite advances in systemic therapies, BC remains a major clinical challenge [2–4]. Among the various sites of metastasis, the brain involvement is particularly concerning due to its significant impact on morbidity and mortality [5]. Depending on the BC subtype, the incidence of brain metastases BM [6] varies between 15 and 50% [7–11]. 

The median overall survival (OS) after BCBM surgery ranges between 6 and 18 months, depending on various factors such as the number of metastases, the extent of systemic disease and the molecular subtype of the primary tumor [12, 13]. Multiple BM are often found in patients with long history of BC and other distant metastasis. Due to their diffuse nature and the limitations of both local and systemic treatment, multiple BM need interdisciplinary and individual treatment concepts [14, 15]. 

The microsurgical resection of a single BM is a widely acknowledged and effective treatment option in the oncological care of BC patients [16]. At the same time, the advisability of surgical treatment for patients with multiple BM remains controversial [15, 17, 18]. It has already been shown in non-small cell lung cancer (NSCLC) that resection may be desirable for multiple BM [19]. However, data on the value of neurosurgical treatment of multiple BCBM is sparse. Since the histological evaluation of metastases helps tailor systemic therapy to the individual receptor status of BC patients, BM resection may prolong survival even in cases of multiple BM [15, 20–26]. 

Therefore, this study aimed to analyze the outcomes of individuals with multiple BCBM who underwent surgical resection of BM at our institution. Additionally, we compared the baseline characteristics and treatment outcomes of patients with single and multiple BCBM after metastasectomy.

## Materials and methods

This study was performed in accordance with the Declaration of Helsinki and approved by the local ethics committee of the University Hospital Essen (local registration number: 17-7855-BO).

### Patient population

All metachronous metastasized female patients (age ≥ 18 years) with resected (single and multiple) BCBM in our institution between January 2008 and December 2019 were included. The selection process of individuals for BCBM surgery within the institutional interdisciplinary neuro-oncologic tumor board was reported previously [27, 28]. The decision regarding postoperative adjuvant radio- or radiochemotherapy, as well as its timing, was based on the recommendations of our institutional tumor board and carefully considered the patients’ clinical condition in the postoperative course. Patients with synchronous cerebral metastases were excluded from the study to analyze a more homogeneous study cohort. In the case of multiple BM, often only one metastasis or several were removed. In individual cases, all BM were resected.

### Data management

Data were collected from the institutional BM database and patients’ electronic medical records, including age, medical history, and specific laboratory parameters at admission to assess anemia (hemoglobin), renal function (creatinine), and inflammatory status (white blood cells). Additionally, BC characteristics were recorded, including the time of BC diagnosis, type of surgical and (neo)adjuvant treatment, histopathological features (invasive ductal, invasive lobular), tumor stage, and receptor status (RS). BM-related parameters included the time of BM diagnosis, preoperative Karnofsky Performance Status (KPS) scale, number and location of BM, RS, and radiographic features on preoperative magnetic resonance imaging (MRI) as previously reported [28, 29]. In addition, all available follow-up data after the BM surgery were collected to evaluate patient survival.

### Statistical analysis

Data were analyzed using SPSS (version 29, SPSS Inc., IBM, Chicago, IL, USA) statistical software. The variables were reported in median values and interquartile ranges (IQR) between 25% and 75%, or as the number of cases (with percentage), as appropriate. The significance level for the p-value was set at ≤ 0.05.

One of the study goals was the comparison of the baseline characteristics and postoperative OS in individuals with single and multiple BM of BC. The preoperative parameters of the sub-cohorts were compared in a univariate analysis using the chi-square test (χ2 test) or the Fisher exact test. The significant results were selected for the final multivariable binary logistic regression analysis to identify independent associations with the presence of single or multiple BM.

The correlations between the clinical characteristics and the OS after multiple BM surgery were tested in the univariable and multivariable Cox regression analysis. We also visualized the survival differences using the Kaplan-Meier survival diagrams and the log-rank test.

**Fig. 1:**
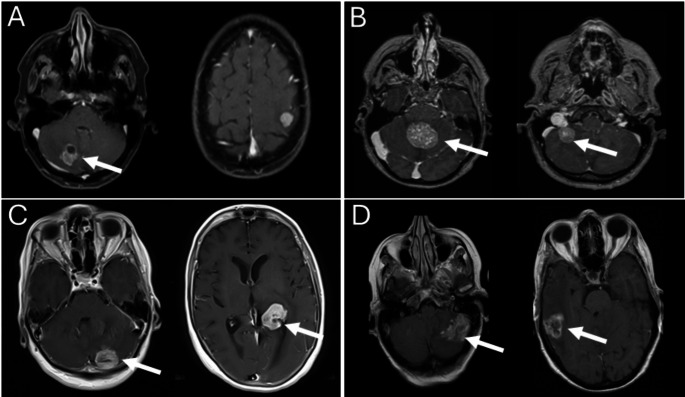
Preoperative MRI (T1 weighted, with contrast agent) of BC patients with multiple BM. **A**: Resection of 1 cerebellar BM, **B**: resection of 2 cerebellar BM, **C**: one stage operation with two craniotomies for resection the cerebellar and the ventricular infiltrated BM, **D**: two-staged operation, first with the resection of a cerebellar BM, followed by a second surgery in 6 months for the resection of the temporal BM. The white arrow shows the metastasis that was/were removed. Abbreviations: BC: breast cancer, BM: brain metastases, MRI: Magnetic Resonance Imaging

Finally, we performed additional analyses comparing the characteristics and OS data in three small subgroups depending on the number and timing of the operated metastases: patients with single (Subgroup 1) and with multiple BM where only one (Subgroup 2) or more than one (Subgroup 3) of metastases was/were operated. The various surgical scenarios for multiple BM depending on the locations and mass-effect of BM are demonstrated in Fig. 1.

## Results

**Fig. 2:**
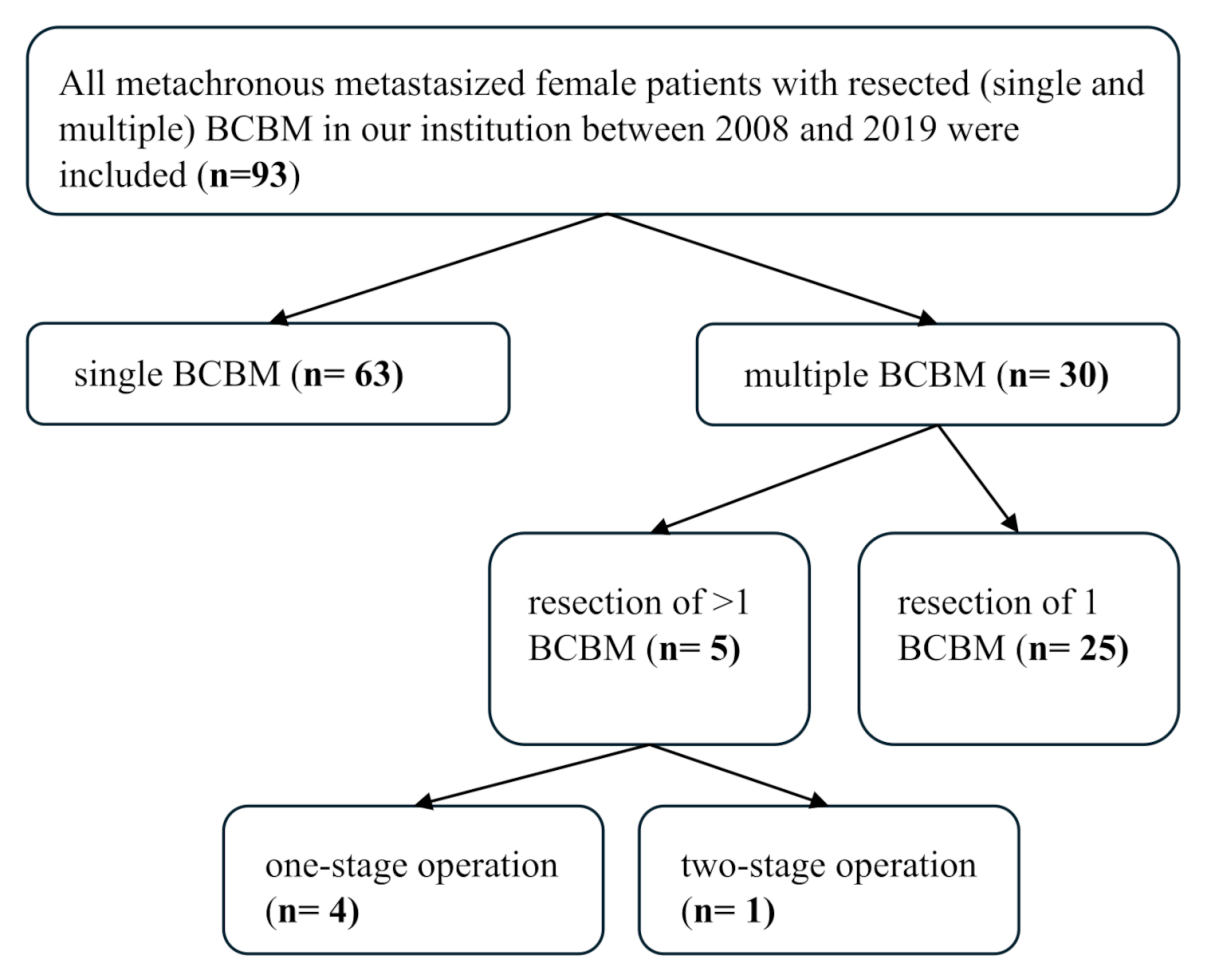
A flowchart showing an overview of the patient cohort. Abbreviations: BCBM: breast cancer brain metastases

The final cohort consisted of 93 patients with metachronous single (*n* = 63) or multiple (*n* = 30) BM (see Flowchart in Fig. 2). For multiple BM, the distribution of the number of cerebral metastases was as follows: two BM *n* = 17, three BM *n* = 6, four BM *n* = 4, six BM *n* = 1, seven BM *n* = 1, nine BM *n* = 1. The median age of the whole cohort at the time of BC diagnosis was 52.0 years (IQR 45.5–62.5). Patient characteristics are summarized in Table 1.

**Table 1:** Baseline characteristics of patients with multiple BCBM

*Parameter*		*median (IQR) or No. (%)*
***BC characteristics***		
Age at BC diagnosis [years]		50.0 (43.8–60.3)
Surgical treatment of BC	*Mastectomy / BPS*	15 (50.0%) / 15 (50.0%)
Systemic treatment of BC	*(Neo-) adjuvant Trastuzumab therapy / without Trastuzumab*	11 (36.7%) / 19 (63.3%)
	*Adjuvant BC radiation / without BC radiation*	18 (60.0%) / 12 (40.0%)
	*Adjuvant Tamoxifen therapy/ without Tamoxifen therapy*	3 (10.0%) / 27 (90.0%)
Histopathology of BC	*Invasive ductal*	17 (56.7%)
	*Invasive lobular*	3 (10.0%)
	*N.a.*	10 (33.3%)
TNM stage	*Initial T stage > T1*	9 (30.0%)
	*Initial N stage ≥ N1*	7 (23.3%)
	*Initial M stage M1*	3 (10.0%)
	*N.a.*	11 (36.7%)
G stage G3	*G3*	9 (30.0%)
	*G2*	6 (20.0%)
	*G1*	1 (3.3%)
	*N.a.*	14 (46.7%)
UICC stage	I-II	15 (50.0%)
	III-IV	6 (20.0%)
	N.a.	9 (30.0%)
BC subtypes	*Basal (= triple-negative)*	5 (16.7%)
	*LumA (HER2-ER + PR+)*	6 (20.0%)
	*LumB (= triple positive)*	4 (13.3%)
	*HER2 (HER2 + ER-PR-)*	11 (36.7%)
	*N.a.*	4 (13.3%)
BC receptor status	*HER2 status positive/ negative*	15 (50.0%) / 11 (36.7%)
	*ER status positive/ negative*	17 (56.7%) / 9 (30.0%)
	*PR status positive/ negative*	10 (33.3%) / 16 (53.3%)
	*N.a.*	4 (13.3%)
***Clinical characteristics***		
Interval BC to multiple BM [months]		42.0 (20.8-131.5)
Age at BM diagnosis [years]		59.5 (51.8–67.0)
Preoperative seizures / not available		0 (0.0%)/ 0 (0.0%)
Preoperative KPS score	≥ 90%	16 (53.3%)
	< 90%	14 (46.7%)
Preoperative laboratory values	*WBC (≥ 10/nl / <10/nl / n.a.)*	14 (46.7%) / 14 (46.7%)/ 2(6.7%)
	*Hemoglobin (< 12g/dl / ≥12g/dl/ n.a.)*	5 (16.7%) / 23 (76.7%)/ 2 (6.7%)
	*LDH (> 247U/l / ≤247U/l/ n.a.)*	11 (36.7%)/ 13 (43.3%)/ 6 (20.0%)
	*GOT (≥ 35U/l/ < 35U/l/ n.a.)*	2 (6.7%)/ 25 (83.3%)/ 3 (10.0%)
	*Creatinine (> 1.1mg/dl / ≤1.1mg/dl/ n.a.)*	0 (0.0%)/ 28 (93.3%)/ 2 (6.7%)
Pre-existing conditions	*Arterial hypertension/ no arterial hypertension*	16 (53.3%)/ 14 (46.7%)
	*Diabetes mellitus/ no diabetes mellitus*	1 (3.3%)/ 29 (96.7%)
	*Hyperuricemia / no hyperuricemia*	0 (0.0%) / 30 (100.0%)
***BM characteristics***		
Preoperative MRI	*Tumor necrosis/ no tumor necrosis/ n.a.*	18 (60.0%) /11 (36.7%)/ 1 (3.3%)
	*Edema (> 10mm/ <10mm/ n.a.)*	24 (80.0%)/ 5 (16.7%)/ 1 (3.3%)
	*Midline shift*	3 (10.0%) /26 (86.7%)/ 1 (3.3%)
	*Supratentorial / infratentorial BM*	12 (40.0%) / 18 (60.0%)
BM receptor status	*HER2 status positive/ negative*	15 (50.0%) / 15 (50.0%)
	*ER status positive/ negative*	14 (46.7%) / 16 (53.3%)
	*PR status positive/ negative*	2 (6.7%) / 28 (93.3%)
	Identic/ converted HER2/ n.a.	23 (76.7%) / 3 (10.0%)/ 4 (13.3%)
	Identic/ converted ER/ n.a.	22 (73.3%) / 4 (13.3%)/ 4 (13.3%)
	Identic/ converted PR/ n.a.	17 (56.7%) / 9 (30.0%)/ 4 (13.3%)

*Abbreviations*: No.: number of cases, IQR: interquartile ranges 25%-75%, OS: overall survival, BC: breast cancer, BM: brain metastasis, HER2: human epidermal growth factor receptor 2, ER: estrogen receptor, PR: progesterone receptor, preop.: preoperative, T: tumor size, N: lymph nodes, M: distant metastasis, G: grade of cancer cells, BPS: breast-preserving surgery, KPS: Karnofsky Performance Score, WBC: white blood cells, UICC: Union for international cancer control, MRI: Magnetic resonance imaging, n.a.: not available, LDH: Lactate dehydrogenase, GOT: Glutamate oxaloacetate transaminase

### Comparison of baseline characteristics between singular and multiple BM

In the univariate analysis of the baseline characteristics, the following parameters were associated with multiple BM: negative PR (progesterone receptor) status in BM (*p* = 0.016), infratentorial tumor localization (*p* = 0.002), hepatic metastasis (*p* = 0.020) and positive human epidermal growth factor receptor 2 (HER2) status in BC (*p* = 0.012, see Supplementary Table 1). In the multivariate analysis, the presence of hepatic metastases (adjusted odds ratio [aOR] 5.86, 95% confidence interval [CI] 1.34–25.61, *p* = 0.019), infratentorial location of resected BM (aOR 3.35, 95% CI 1.03–10.83, *p* = 0.044), and a positive HER2 RS in the primary BC (aOR 3.93, 95% CI 1.23–12.53, *p* = 0.021) were more common in the cohort with multiple BM (see Supplementary Table 2).

### Prognostic factors for OS after BM surgery in multiple BM

Adjuvant radiotherapy was administered to 25 patients with multiple BM, including 14 who received stereotactic radiotherapy and 11 who underwent whole-brain radiotherapy. Of the remaining patients, two did not receive postoperative irradiation, and data on adjuvant treatment were unavailable for three cases.

**Fig. 3:**
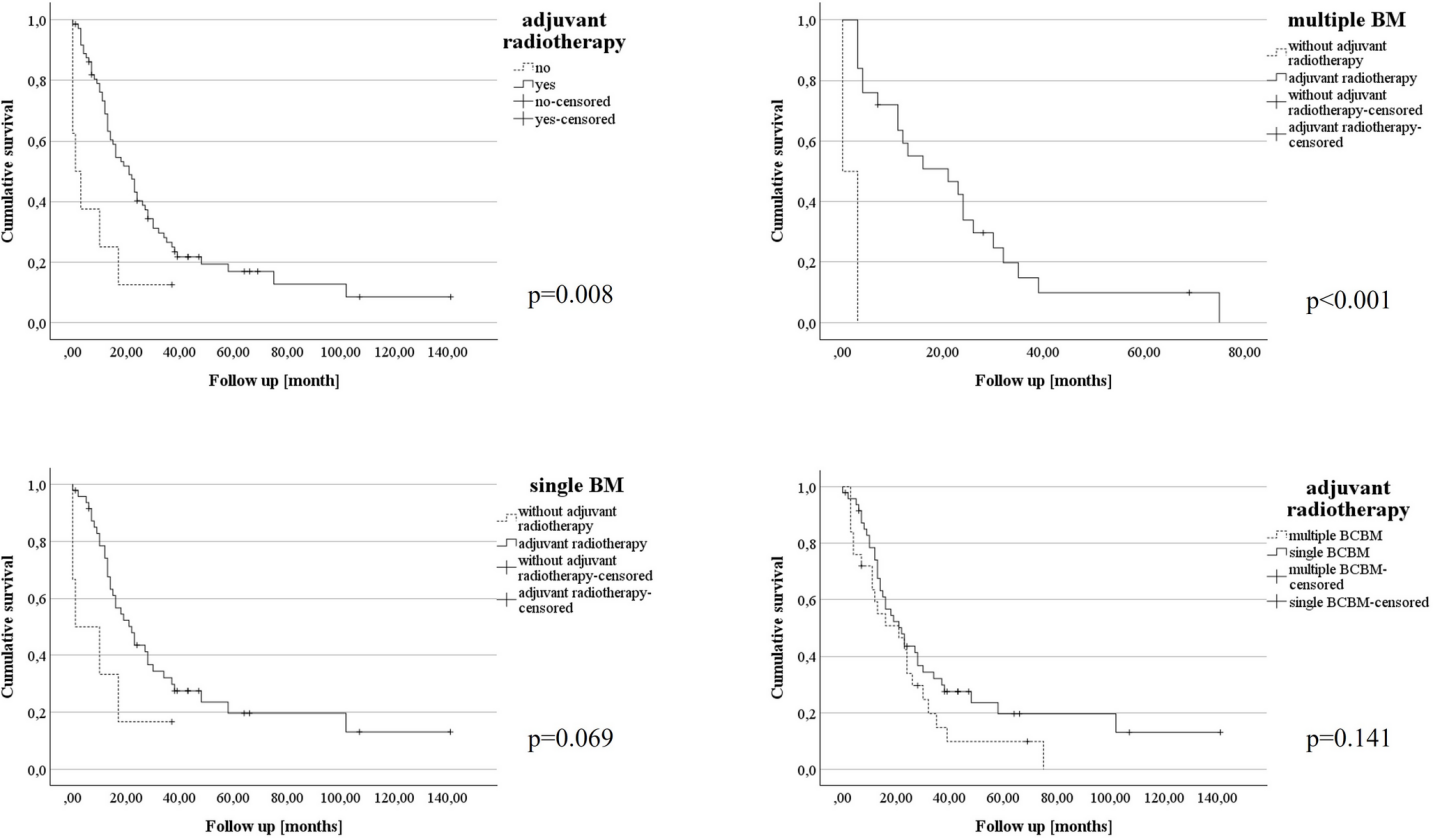
Adjuvant radiotherapy improved survival after BM resection in BCBM with multiple BM. **A**: the whole cohort (single and multiple BCBM), **B**: multiple BCBM patients, **C**: single BCBM patients, **D**: comparison between single and multiple BCBM patients after adjuvant radiotherapy. Abbreviations: BM: brain metastases, BCBM: breast cancer brain metastases

The univariate Cox regression analysis demonstrated adjuvant radiotherapy (*p* = 0.006), adjuvant BC trastuzumab therapy (*p* = 0.001) and the supratentorial location (*p* = 0.011) as significant prognostic factors for survival in multiple BM patients (see Supplementary Table 3). The final multivariate Cox regression analysis confirmed the importance of adjuvant radiotherapy of BM (adjusted hazard ratio [aHR] 5.93, 95% CI 1.06–33.26, *p* = 0.043) and the chemotherapy with trastuzumab after BC (aHR 4.95, 95% CI 1.72–14.25, *p* = 0.003) for OS after metastasectomy of multiple BM patients (see Table 2). The Kaplan-Meier survival curves illustrate a positive effect of adjuvant radiotherapy after metastasectomy (Fig. 3A), especially for multiple BCBM (see Fig. 3B). This effect was less pronounced in patients with single BM (see Fig. 3C). In the sub-population of individuals with postoperative radiotherapy, the comparison between single and multiple BCBM showed no significant difference (see Fig. 3D).

**Table 2:** Multivariate Cox regression analysis of postoperative survival after BM surgery in BCBM patients with multiple BM (*n* = 30)

Multivariable cox regression analysis			
Parameter	*p*-value	aHR	95% CI
adjuvant radiotherapy	0.043	5.93	1.06–33.26
supratentorial BM	0.069	2.37	0.93–6.02
adjuvant Trastuzumab	0.003	4.95	1.72–14.25

Abbreviations: BM: brain metastasis, BC: breast cancer, aHR: adjusted hazard ratio, CI: confidence interval, BCBM: breast cancer brain metastasis

### Comparison of OS in different subgroups

The OS analysis shows a median survival of 17.0 months (IQR 9.0–37.0) for single BM and 12.5 months (IQR 3.75–28.5, *p* = 0.271) for multiple BM. When comparing the group of multiple BMs in which one BM was resected with the group with more than one resected BM, the median OS results are as follows: 13.0 months (IQR4.0-26.0) versus 7.0 months (IQR3.00-30.00, *p* = 0.557).

The Kaplan Meier survival plot did not show a significant difference in OS between subgroups with single and multiple BM (*p* = 0.186, see Supplementary Fig. 1). Further stratification of multiple BM cases did not reveal significant survival differences depending on the number of resected metastases as well (*p* = 0.972, see Supplementary Fig. 2).

### Functional outcome after BM surgery in multiple BM patients

Our data shows an improvement in the neurological deficits or, at least, a stable neurological status in 83.3% (*n* = 25) of patients after surgery. At the end of hospital treatment, 80% of cases in the cohort (*n* = 24) showed a KPS of above 70% (see Supplementary Table 4).

## Discussion

Individualized treatment strategies can improve the prognosis of patients with multiple BCBM. The number of BM can vary and patients with a long history of BC often develop multiple BM, challenging the treatment decision in these patients. Our findings justify the surgical resection of one or more BM in selected cases presenting with multiple BCBM.

Commonly, indication on BM resection depends on the size and clinical relevance of metastatic lesion(s) [30, 31]. The decision to treat multiple BM surgically is controversial [15, 17, 18, 23]. There are opinions that multiple BM are not eligible for resection or that the outcome is significantly worse than with single BM [15, 30]. On the other hand, there are also reports showing that with controlled extracranial disease, patients with multiple BM can benefit from surgery [19, 23]. More recent studies also confirmed a positive effect of surgery on survival in case of multiple BM [17, 26, 32]. Moreover, Bschorer et al. were able to show that patients with different primary cancers can also benefit from the resection of multiple BM, even if this requires several craniotomies [26]. Our results support the surgical treatment of multiple BM, as postoperative overall survival in these patients was comparable to that of those with a single BM. We were moreover able to emphasize in our larger, more homogeneous cohort of BC patients that surgical treatment is recommended for multiple BM.

Of note, surgical treatment is not always feasible in cases of multiple BM, placing other methods of local treatment at the forefront. In particular, the whole brain radiotherapy is a common and effective treatment option for patients with multiple BM [33, 34]. Other clinical studies demonstrated a good local control with lower toxicity after stereotactic radiosurgery (SRS) [35]. When comparing SRS with surgical resection in individuals with multiple BCBM, studies reported the equal effects on local tumor control and patients’ survival [36]. 

When evaluating the advisability of surgical intervention in patients with multiple BM, the potential oncological benefit of analyzing metastasis samples should also be considered. This diagnostic insight can help optimize subsequent oncological treatment and potentially improve disease prognosis [17]. This circumstance is of particular relevance for BC patients, as previous studies have shown that receptor conversion can occur over time, necessitating the diagnostic reevaluation of metastases for optimization of systemic treatment [27, 37]. In their series on surgically treated patients with multiple BM, Ersoy et al. demonstrated a significantly better prognosis in individuals with BC than in those with other primary tumors [17]. This finding underscores the potential value of surgical therapy in patients with BCBM, even in the setting of multiple BM. Beyond its therapeutic role, surgery facilitates the acquisition of metastatic tissue for molecular diagnostics, enabling precise treatment adjustments tailored to the evolving tumor profile.

Regarding survival predictors after surgery for multiple BCBM, our study identified trastuzumab as being significantly associated with improved postoperative prognosis. However, there are conflicting views on the role of trastuzumab, which is a well-established adjuvant therapy for HER2-positive BC [38]. Notably, patients with HER2-positive BC have an increased risk of developing BM [13]. It is important to recognize that trastuzumab does not readily penetrate the central nervous system (CNS) when the blood-brain barrier is intact, despite demonstrating strong efficacy against extracranial metastases [39, 40]. Consequently, trastuzumab therapy may reduce the occurrence of extracranial metastases, potentially leading to CNS metastases being detected as the first and sometimes sole sites of disease progression [13, 40]. Interestingly, a large study published in 2020 demonstrated that adjuvant trastuzumab treatment significantly improved progression-free survival in BC patients with BM [41]. Our findings further support the advisability of trastuzumab as an adjuvant treatment following surgery for multiple BM in BC patients.

In summary, an individualized approach is crucial when selecting the optimal local treatment for patients with multiple BM. Key factors influencing treatment decisions include the patient’s overall clinical condition, age, and oncological disease stage, as well as the anatomical characteristics and operability of the metastases. Our findings, along with data from the literature, suggest that surgical treatment may be a viable option for selected patients with multiple BM, at least for a subset of their metastases. Beyond the symptomatic relief that surgery can provide, the value of obtaining tumor samples for molecular and histopathological reevaluation should not be underestimated. Given that RS conversion after initial treatment is not uncommon in BC patients, the diagnostic insights gained from BM samples enable a more precise refinement of systemic therapy, which is essential for optimizing prognosis. This highlights the critical role of surgery in BM management, even for patients with multiple lesions.

### Limitations

Limitations of our study include the monocentric retrospective design, relatively small sample size and incomplete follow-up data. Secondly, only neurosurgically treated BCBM patients were included. Therefore, a comparison with non-surgically treated multiple BM cases is missing. The analysis regarding perioperative safety and postoperative neurological outcome is missing in the current analysis and will be included in future analyses. Finally, individuals with multiple BM constitute a heterogeneous patient group, as evidenced by the various surgical strategies employed within this subgroup (see Fig. 1). To address the major limitations of the present study, we suggest a multicenter study involving larger patient samples that are not limited to neurosurgical cases.

## Conclusion

With adequate patient selection through an interdisciplinary tumor board, patients with multiple BCBM selected for BM resection have outcomes comparable to those with singular BCBM. Our data support the surgical treatment of multiple BCBM in patients with appropriate clinical conditions and accessible BM locations.

## Electronic supplementary material

Below is the link to the electronic supplementary material.

Supplementary Material 1

## Data Availability

Data is provided within the manuscript or supplementary information files.
