# Supplementary material for: Surgical treatment of multiple breast cancer brain metastases: clinical characteristics and factors impacting postoperative survival
Source: J Neurooncol. 2025 Apr 29;174(1):157–65. doi: 10.1007/s11060-025-05048-3 (PMC12198272; doi:10.1007/s11060-025-05048-3)
Supplement: Supplementary file 1 — Supplementary Material 1 [file 11060_2025_5048_MOESM1_ESM.docx]

**Journal of Neuro-Oncology**

**Surgical treatment of multiple breast cancer brain metastases: clinical characteristics and factors impacting postoperative survival**

*Anna Michel^1,2,3^, Laurèl Rauschenbach^1,2,3,4^, Hanah Karadachi^1,2,3^, Meltem Gümüs^1,2,3^, Yahya Ahmadipour^1,2,3^, Marvin Darkwah Oppong^1,2,3^, Christoph Pöttgen^3,5^, Jörg Hense^,3,6^, Neriman Özkan^1,2,3^, Karsten H. Wrede^1,2,3^, Philip Dammann^1,2,3^, Ulrich Sure^1,2,3^, Ramazan Jabbarli ^1,2,3^*

**Affiliation:**

1. Department of Neurosurgery and Spine Surgery, University Hospital Essen, Essen, Germany
2. Center for Translational Neuro‑ & Behavioral Sciences (C‑TNBS), University Duisburg Essen, Essen, Germany
3. German Cancer Consortium (DKTK) Partner Site, University Hospital Essen, 45147 Essen, Germany
4. German Cancer Research Center (DKFZ) Division Translational Neurooncology at the West German Cancer Center (WTZ), DKTK Partner Site, University Hospital Essen, Essen, Germany
5. Department of Radiotherapy, University Hospital Essen, Essen, Germany
6. Department of Medical Oncology, University Hospital Essen, Essen, Germany

**Corresponding author:**

Dr. med. Anna Michel

Department of Neurosurgery and Spine surgery, University Hospital Essen, University Duisburg-Essen, Hufelandstraße 55, 45147 Essen, Germany

E-Mail: anna.michel@uk-essen.de

Telephone Number: 00492017231230/ Fax Number: 00492017231220

**SUPPLEMENTARY MATERIAL**

**Supplementary Figure 1** Kaplan Meier and Cox Regression Analysis of postoperative survival after brain metastasis surgery. No significant difference in postoperative survival between individuals with multiple (median: 12.5 months) and single (median: 17.0 months) BM, p=0.186.


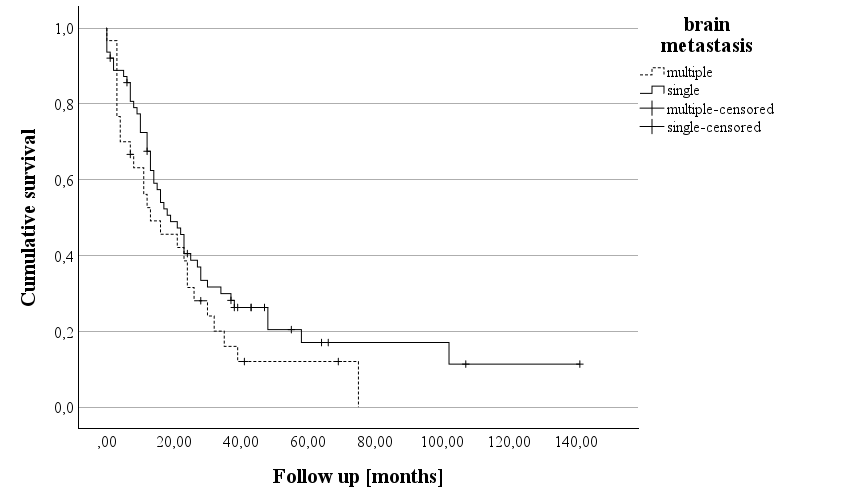


**Supplementary Figure 2** Kaplan Meier curve demonstrated postoperative survival after BCBM surgery for three subgroups (subgroup 1= operated single BCBM (n=63), subgroup 2 = one operated metastasis in patients with multiple metastases (n=25), subgroup 3 = more than one operated metastasis in patients with multiple metastases (n=5)).


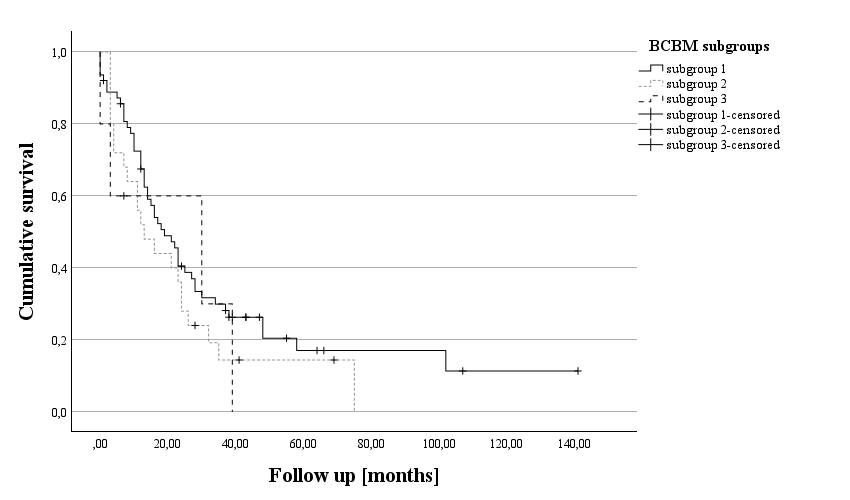


p=0.200

Abbreviation: BCBM: breast cancer brain metastases

**Supplementary Table 1** Univariate analysis (chi-square test) of predictors for multiple BCBM

| **Parameter** | **p-value** | **OR** | **95% CI** |
| --- | --- | --- | --- |
| **BC related parameters** |  |  |  |
| age at BC diagnosis <60 years | 0.340 | 1.18 | 0.90-1.54 |
| invasive ductal BC | 1.000 | 1.02 | 0.81-1.28 |
| pos. HER2 BC | 0.012 | 2.18 | 1.25-3.81 |
| **BM related parameter** |  |  |  |
| age at BM diagnosis <65 years | 0.497 | 1.17 | 0.84-1.63 |
| hepatic metastasis | 0.020 | 2.95 | 1.25-7.00 |
| infratentorial BM | 0.002 | 2.36 | 1.41-3.95 |
| neg.PR BM | 0.016 | 1.31 | 1.09-1.57 |
| adjuvant radiotherapy | 0.712 | 1.04 | 0.90-1.20 |
| adjuvant Trastuzumab | 0.210 | 0.21 | 0.85-3.19 |
| preoperative KPS>80 | 0.722 | 0.96 | 0.82-1.13 |
| extracranial metastases | 0.042 | 1.74 | 1.05-2.88 |
| dural attachment | 0.358 | 1.26 | 0.86-1.87 |
| MRI cystic component | 0.798 | 1.10 | 0.53-2.31 |
| MRI with necrotic area | 0.364 | 1.22 | 0.83-1.79 |
| Converted RS | 0.628 | 1.19 | 0.76-1.86 |

Abbreviations: BC: breast cancer, BM: brain metastasis, HER2: human epidermal growth factor receptor 2, PR: progesterone receptor, preop.: preoperative, KPS: Karnofsky Performance Score, MRI: Magnetic resonance imaging, pos.: positive, neg.: negative, RS: receptor status, OR: odds ratio. CI: confidence interval

**Supplementary Table 2** Binary logistic multivariable analysis of baseline characteristics and of prognostic factors for patients with multiple BM compared to single BM. (n=93)

| **Multivariable binary logistic regression analysis** | | | |
| --- | --- | --- | --- |
| **Parameter** | **p-value** | **aOR** | **95% CI** |
| hepatic metastasis | 0.019 | 5.86 | 1.34-25.61 |
| infratentorial BM | 0.044 | 3.35 | 1.03-10.83 |
| pos. HER2 BC | 0.021 | 3.93 | 1.23-12.85 |
| neg.PR BM | 0.101 | 4.00 | 0.76-20.85 |

Abbreviations: BM: brain metastasis, BC: breast cancer, pos.: positive, neg.: negative, HER2: human epidermal growth factor receptor 2, PR: progesterone receptor, aOR: adjusted odds ratio. CI: confidence interval

**Supplementary Table 3** Univariable cox regression analysis of prognostic factors for patients with multiple BM

| **Parameter** | **p-value** | **HR** | **95% CI** |
| --- | --- | --- | --- |
| **BC related parameters** |  |  |  |
| age at BC diagnosis <60 years | 0.491 | 1.43 | 0.52-3.96 |
| invasive ductal BC | 0.132 | 3.54 | 0.68-18.38 |
| pos. HER2 BC | 0.078 | 2.25 | 0.91-5.53 |
| **BM related parameter** |  |  |  |
| age at BM diagnosis <65 years | 0.471 | 1.36 | 0.59-3.09 |
| without extracranial metastases | 0.313 | 1.51 | 0.68-3.39 |
| dural attachment | 0.963 | 1.02 | 0.44-2.34 |
| MRI cystic component | 0.909 | 1.05 | 0.45-2.47 |
| MRI without necrotic area | 0.461 | 1.38 | 0.59-3.24 |
| Identic RS | 0.647 | 1.23 | 0.50-3.02 |
| BM resection number ≤1 | 0.972 | 1.02 | 0.347-2.99 |
| hepatic metastasis | 0.966 | 1.02 | 0.45-2.32 |
| adjuvant BM radiotherapy | 0.006 | 11.04 | 1.99-61.11 |
| supratentorial BM | 0.011 | 3.10 | 1.30-7.40 |
| adjuvant Trastuzumab | 0.001 | 6.11 | 2.20-16.98 |
| preop. KPS >80 | 0.472 | 1.58 | 0.45-5.54 |
| Pos. PR BM | 0.574 | 1.52 | 0.35-6.53 |

Abbreviations: BC: breast cancer, BM: brain metastasis, HER2: human epidermal growth factor receptor 2, PR: progesterone receptor, preop.: preoperative, KPS: Karnofsky Performance Score, MRI: Magnetic resonance imaging, pos.: positive, neg.: negative, RS: receptor status, HR: hazard ratio, CI: confidence interval

**Supplementary Table 4** Postoperative neurological outcomes in multiple BCBM patients

| **Parameter** |  | **No. (%)** |
| --- | --- | --- |
| Preoperative focal neurological deficit | Worse/ stable/ improved | 5 (16.7%)/ 20 (66.7%)/ 5 (16.7%) |
| Motor deficit | No/ yes | 29 (93.3%)/ 2 (6.7%) |
| Language deficit | No/ yes | 28 (29.3%)/ 2 (6.7%) |
| Postoperative KPS | ≥80%/ <80% | 24 (80.0%)/ 6 (20.0%) |

Abbreviations: KPS: Karnofsky Performance Score
